# Comparative Genomics of *Halobacterium salinarum* Strains Isolated from Salted Foods Reveals Protechnological Genes for Food Applications

**DOI:** 10.3390/microorganisms11030587

**Published:** 2023-02-25

**Authors:** Alessandra Fontana, Irene Falasconi, Paolo Bellassi, Elisabetta Fanfoni, Edoardo Puglisi, Lorenzo Morelli

**Affiliations:** 1 Department for Sustainable Food Process—DiSTAS, Università Cattolica del Sacro Cuore, Via Bissolati, 74, 26100 Cremona, Italy; 2 Department for Sustainable Food Process—DiSTAS, Università Cattolica del Sacro Cuore, Via Emilia Parmense, 84, 29122 Piacenza, Italy

**Keywords:** archaea, *Halobacterium salinarum*, *Halobacteria*, halophilic, salted food, comparative genomics, functional food, food technology, halocins

## Abstract

Archaeal cell factories are becoming of great interest given their ability to produce a broad range of value-added compounds. Moreover, the Archaea domain often includes extremophilic microorganisms, facilitating their cultivation at the industrial level under nonsterile conditions. Halophilic archaea are studied for their ability to grow in environments with high NaCl concentrations. In this study, nine strains of *Halobacterium salinarum* were isolated from three different types of salted food, sausage casings, salted codfish, and bacon, and their genomes were sequenced along with the genome of the collection strain CECT 395. A comparative genomic analysis was performed on these newly sequenced genomes and the publicly available ones for a total of 19 *H. salinarum* strains. We elucidated the presence of unique gene clusters of the species in relation to the different ecological niches of isolation (salted foods, animal hides, and solar saltern sediments). Moreover, genome mining at the single-strain level highlighted the metabolic potential of *H. salinarum* UC4242, which revealed the presence of different protechnological genes (vitamins and *myo*-inositol biosynthetic pathways, aroma- and texture-related features, and antimicrobial compounds). Despite the presence of genes of potential concern (e.g., those involved in biogenic amine production), all the food isolates presented archaeocin-related genes (halocin-C8 and sactipeptides).

## 1. Introduction

Halophilic archaea are studied for their ability to grow in environments with high NaCl concentrations. In particular, the class *Halobacteria* comprises microorganisms that require a NaCl concentration of at least 1.5 M for proliferation and that show optimal growth at 3.5–4.5 M [1]. Its members are ubiquitous in environments containing NaCl concentrations up to saturation, such as solar salterns and soda lakes [2,3,4], but can also live in marine environments and in salted food products [5,6,7,8]. The vast majority of the known microorganisms cannot proliferate at water activity (a_w_) values below 0.90 [9]. However, the most extremophilic species can undergo cell division at a_w_ down to 0.61; indeed, some xerophilic fungi can grow and/or germinate within a a_w_ range of 0.755–0.605 [10]. The ability of extreme halophiles to grow at a_w_ values such as that of saturated NaCl relies on different adaptation strategies to balance cellular osmotic stress [11,12]. For instance, halophilic archaea counteract the external low a_w_ by accumulating K^+^ and, in some cases, Na^+^ ions [13]. Moreover, the enzymes of these halophiles can work at high NaCl concentrations since their proteins include a high content of acidic amino acids (i.e., glutamate and aspartate). To this extent, the first sequenced genome *Halobacterium* sp. NRC-1 allowed researchers to explore more in depth the mechanisms behind its ecological adaptation to hypersaline brine. Genotypic characterization indeed revealed that most of the predicted proteins were highly acidic and that the negatively charged residues were mostly located at the protein surface. This feature was associated with the function of increasing their solubility and stability in high-salt-concentration environments, making halophile enzymes active at a_w_ values equal to or below 0.755 [11,14,15,16]. The neutron spectroscopy of the proteome of *Halobacterium salinarum* showed that protein structure is strongly affected by high NaCl since K^+^ concentrations below 2.2 M induce their misfolding and precipitation [17].

Haloarchaea are mostly aerobes, but a few species can grow anaerobically by using arginine and nitrate as terminal electron acceptors [1]. Halophilic members were initially found to be spoilage microorganisms in salted products, such as salted codfish and brine-cured hides in the leather industry. However, they have also been utilized in food processing as ameliorating microorganisms; for instance, they have been exploited to accelerate fish sauce fermentation [18] and to improve the safety and quality of salted anchovies [19]. Indeed, it has been highlighted that salted anchovies inoculated with halophiles showed lower loads of naturally present staphylococci, *Enterobacteriaceae*, and lactic acid bacteria. Moreover, a decrease in the histamine content and an improvement in the organoleptic characteristics have also been evidenced [19]. Other than this, several studies have been carried out to assess how halophilic archaea could play a key role in the fermentation process of fish products, showing that this last role is promoted by specific enzymes, such as serine proteases, that can be active even in environments characterized by high NaCl concentrations [20]. The most well-studied member of the class *Halobacteria* is *Halobacterium salinarum*, which is the type species of the genus *Halobacterium*, and it was first isolated from salted codfish [21,22]. Since their discovery, halophilic archaea have attracted great scientific interest mainly due to their extraordinary ability to grow at high salinity levels, prompting many studies focusing on these microorganisms’ proteins, lipids, and enzymes [16]. Indeed, most of their proteins and enzymes maintain functionality even under the presence of 10–35% NaCl and under temperatures up to 100 °C at which most bacterial proteins become denatured and/or nonfunctional [23]. To reveal the molecular mechanisms behind the physiological response of *Halobacteria* in extreme environments, *H. salinarum* NRC-1 has represented a model microorganism used to elucidate evolutionary adaptation to such conditions (high salinity and low water activity). Indeed, the poor characterization of extreme halophiles can be bypassed with the exploitation of the increasing presence of genome sequences to develop system-level models able to predict regulatory and functional connections between genes and key abiotic factors in hypersaline environments [24].

Currently, archaeal cell factories are becoming of high interest given their abilities in producing a large variety of value-added compounds; moreover, the fact that their members are often extremophilic microorganisms facilitates their cultivation at the industrial level under nonsterile conditions using inexpensive feedstocks [25]. Up to date, commercially available archaeal products are all derived from halophiles and are bacteriorhodopsin, bacterioruberin, diether/tetraether lipids, and squalene [25]. However, other archaeal applications are under investigation at different technology readiness levels (TRLs), such as for the production of carotenoids, polyhydroxyalkanoates, methane, and biohydrogen [25]. Halotolerant microorganisms specifically play an important role in food biotechnology for the production of fermented foods and food supplements. Indeed, they produce different compounds that characterize the food product in terms of aroma, flavor, and appearance as well as in terms of nutritional value (vitamins and derivatives) [25,26]. Furthermore, their capability to produce antimicrobial peptides (i.e., archaeocins) has also been assessed [27]. It is also important to highlight that the tolerance of haloarchaea to environments with low water activity is not only an advantage in the food sector, but it can also be exploited in arid soils used for crop production by mediating the plant–microbe and plant–insect interactions [12].

In this study, we investigated 19 *H. salinarum* strains isolated from different ecological niches (i.e., salted foods, animal hides, and solar saltern sediments) to highlight the specific genetic signatures of adaptation to the different environments. In addition, the genome mining of salted food isolates was further deepened to evaluate the presence of functional and protechnological genes as well as genes of health concern for their possible exploitation as natural starters in salted food products.

## 2. Materials and Methods

### 2.1. Isolation and Molecular Fingerprinting of Halophilic Archaea from Salted Foods

Halophilic archaea were isolated from three different types of salted food: sausage casings, salted codfish, and bacon. A total of 10 grams of each sample were mixed with 90 mL of saline water (NaCl concentration of 200 g/L) and were homogenized for 1.5 min at 260 rpm using a Stomacher machine (400 Circulator; International PBI, Milan, Italy). A 1 mL aliquot of this homogenate was serially diluted in 9 mL of Halobacteria medium (DSM 372; Deutsche Sammlung von Mikroorganismen und Zellkulture GmbH, DSMZ) and was used for microbiological analyses. Sample suspension aliquots of 100 µL were plated on duplicate agar plates made with DSM 372 medium (yeast extract 5 g/L, casamino acids 5 g/L, Na-glutamate 1 g/L, KCl_2_ g/L, Na_3_-citrate 3 g/L, MgSO_4_ × 7 H_2_O 20 g/L, NaCl 200 g/L, FeCl_2_ × 4 H_2_O 0.036 g/L, MnCl_2_ × 4 H_2_O 0.00036 g/L, and agar 20 g/L). The plates were incubated at 37 °C for 2 weeks. Representative colonies were selected depending on color, size, and morphology. DNA was extracted from isolated purified colonies using microLYSIS^®^-Plus (MicroZone Limited) following the manufacturer’s protocol. To identify unique isolates, randomly amplified polymorphic DNA polymerase chain reaction (RAPD-PCR) was performed using GTG-5 (5′-GTGGTGGTGGTGGTG-3′) primer [28]. The PCR fragment profiles were analyzed using Fingerprinting II software (Bio-Rad Laboratories, Hercules, CA, USA). Similarities between band profiles were determined by calculating the Pearson correlation coefficient, and cluster analyses were performed using the provided unweighted pair group method with arithmetic mean (UPGMA). A correlation coefficient of 85% was selected to distinguish the clusters. One representative isolate from each cluster was amplified for 16S rRNA gene using the primers 7F (5′-TTCCGGTTGATCCYGCCRG-3′) and 1492R (5′-TACGGYTACCTTGTTACGACTT-3′) for archaea [29]. Amplified 16S rRNA genes were Sanger sequenced and identified through alignment against the NCBI database.

### 2.2. Whole-Genome Sequencing and Comparative Genomics of Selected Halobacterium salinarum Strains

Nine identified *H. salinarum* unique isolates (three per type of salted food considered) were selected for whole-genome sequencing along with the collection strain CECT 395. Genomic DNA was extracted with the MasterPure™ Gram Positive DNA Purification Kit (Lucigen Corporation, Middleton, WI, USA) according to the provided protocol. The quality of the extracted DNA was then checked by means of agarose gel electrophoresis (0.8%), and the quantity was determined using Qubit fluorometer (Life Technologies, Carlsbad, CA, USA). Whole-genome sequencing was then performed with the Illumina MiSeq platform using the TruSeq Nano Kit (Illumina Inc., San Diego, CA, USA) for library preparation (2 × 150 bp). Quality filtering and adaptor removal were carried out using Trimmomatic software (v0.39) [30]. De novo genome assembling was performed with Unicycler in BV-BRC (v3.27.7) [31]. The quality of the assembled genomes was evaluated with QUAST (v4.6.0) [32], whereas the completeness and contamination levels were estimated with CheckM (v1.2.2) [33]. The taxonomic identification of the genome assemblies was further verified through dDDH calculation using the Type Strain Genome Server [34]. Annotation of the genome assemblies was then carried out with Prokka (1.14.6) [35].

Pangenome analysis was performed using Roary (3.13.0) [36] as previously described [37,38] on the 10 sequenced strains along with the 9 publicly available genomes of *H. salinarum* from the NCBI database (accessed on 30 November 2022) for a total of 19 genome sequences (Table 1). The core gene alignment resulting from Roary was then used in RAxML (8.2.12) to build a maximum likelihood phylogenetic tree. The effect of the isolation source on the genomic content of the strains was evaluated in Past3 (3.26) [39] using a principal coordinates analysis (PCoA) based on Bray–Curtis dissimilarity metrics of the gene presence/absence matrix generated from Roary. Metabolic pathways were analyzed using KEGG annotation within the Comparative Pathway tool in BV-BRC. Bacteriocin-like antimicrobial substances (i.e., archaeocins) were evaluated using BAGEL4 [40]. Antibiotic resistance genes (ARGs) were investigated with the Resistance Gene Identifier (RGI) tool based on CARD Database [41], whereas virulence factor (VF) genes were searched with the Abricate tool (v1.0.1) based on Virulence Factor Database (VFDB) [42].

## 3. Results and Discussion

### 3.1. Molecular Fingerprinting of Halophilic Archaea Isolates

A total of 65 representative colonies were isolated from the 3 types of salted food investigated. Specifically, 46 isolates were obtained from sausage casings, 10 isolates were obtained from salted codfish, and 9 isolates were obtained from bacon. RAPD-PCR fingerprinting allowed us to distinguish 33 different profiles having a maximum similarity index of 85% as determined through UPGMA clustering (Figure S1). The 33 unique strains were subjected to 16S rRNA gene sequencing, revealing their belonging to the *H. salinarum* species (Table S1). From the RAPD-PCR profiles, it was also shown that a clusterization of the isolates originated from the sausage casings, whereas the bacon and salted codfish isolates did not evidence a distinct separation (Figure S1). From the fingerprinting results, three representative unique strains of each isolation source were selected for an in-depth characterization of the strains at the genome level.

### 3.2. Comparative Genomic Analysis of H. salinarum Strains

Considering the general genomic features, based on the isolation source (Table S2), the food strains showed an average genome length of 2.7 Mbp with a 65% GC content, whereas the animal and environmental strains exhibited a slightly shorter genome (2.5 and 2.3 Mbp) and a higher GC (66.1% and 66.2%). The belonging of the genomes to the *H. salinarum* species was additionally confirmed through dDDH (%dDDH > 70%) against the Type Strain Genome Server (Table S3).

A comparative genomic analysis on the 19 *H. salinarum* strains revealed a pangenome of 8430 genes. Specifically, 1101 genes belonged to the core genome (i.e., genes shared between 99% and 100% of the strains); no “soft core” genes (shared between 95% and 98%) were detected, whereas “shell” genes (shared between 15% and 94%) and “cloud” genes (included in less than 15% of the strains) totaled 2285 and 5044 genes, respectively (Table S4). A previous analysis at the class level on the *Halobacteria* pangenome was carried out on 111 genomes belonging to different halobacterial species [43]. The study revealed a core genome of 300 genes, thus being almost 4 times smaller than the pangenome identified for the *H. salinarum* species investigated in this study. This finding could mainly be due to the fact that the *class* taxonomic level represents a wide range of different species which likely share less genes between each other than strains belonging to a single species. Despite this, the higher number of core genes in the *H. salinarum* species compared to other prokaryotes species [44,45] could be mainly due to the limited availability of public *H. salinarum* genomes and, thus, could be due to the limited “biodiversity” considered. This concept was also confirmed by our results, showing that the *H. salinarum* pangenome is still open since an average of 300 new genes were added for each additional genome included in the analysis (Figure S2). Indeed, it has been previously pointed out that the accuracy of the pangenome definition of a given species strongly depends on sampling the broadest genome diversity possible to best define the core genes and phylogenetic relationships between the genomes analyzed [46].

**Figure 1:**
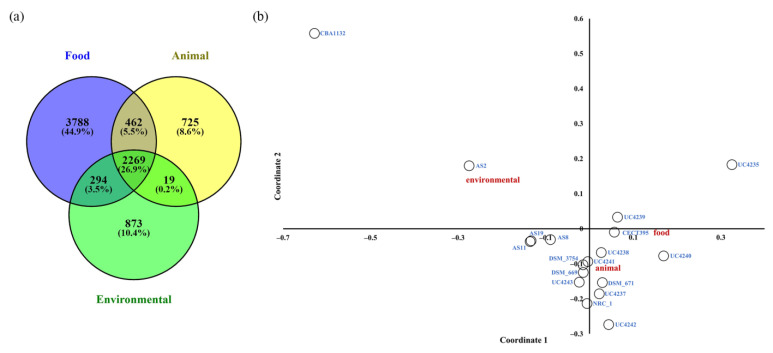
(**a**) Venn diagram representing the percentage of shared and niche-specific genes within *H. salinarum* pangenome. (**b**) PCoA based on the gene presence/absence matrix and isolation source category (labelled as food, animal, and environmental) of the *H. salinarum* strains investigated. The distance between niche labels represents the dissimilarity in the genetic content of the isolates in relation to their isolation source.

The percentage of shared and niche-specific genes within the pangenome of *H. salinarum* was also evaluated (Figure 1a). The Venn diagram shows that approximately 27% of the overall gene content was shared among the three ecological niches from which the strains were isolated. The higher percentage of genes specifically present within the food-isolated genomes could mainly be addressed to the higher number of food strains considered in the analysis (10 out of 19). However, it seemed that the food and animal isolates shared more genes than the latter shared with the environmental isolates. A statistical PCoA based on the pangenome and the isolation source of the strains additionally evidenced the dissimilarity between the genetic contents of the isolates considered (Figure 1b). Indeed, it was shown that the environmental, animal, and food isolates split into different clusters within the two coordinates, revealing the presence of genes specifically related to the ecological niche.

**Figure 2:**
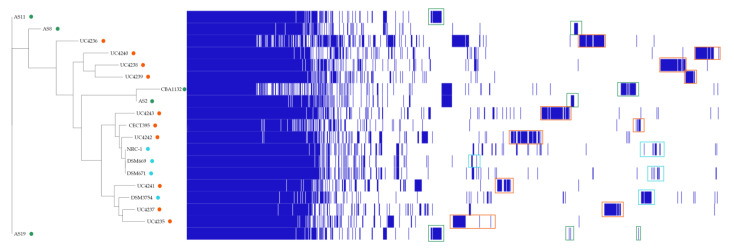
Phylogenetic tree based on the core gene alignment and the gene presence/absence matrix of the investigated strains. Orange, cyan, and green circles represent food, animal, and environmental *H. salinarum* strains, respectively. Orange, cyan, and green rectangles indicate unique gene clusters of each strain in relation to the isolation category.

Based on the presence/absence of core and accessory genes, the Roary matrix highlighted the presence of unique gene clusters in each considered genome (Figure 2). 

**Figure 3:**
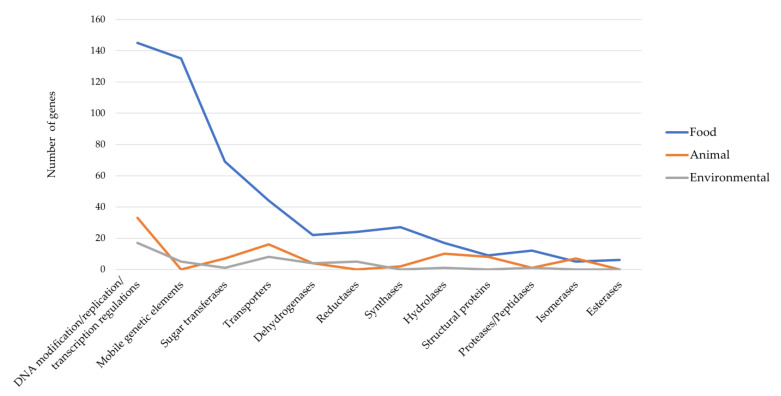
Main categories of genes found within the unique gene cluster present in each strain for the three different ecological niches investigated.

Regarding the food-niche isolates, a total of 2752 unique genes were evidenced. Excluding hypothetical proteins, the categories that had more genes were related to the DNA modification/replication/transcription regulations and to the mobile genetic elements (i.e., transposases, site-specific integrases, recombinases, and insertion sequences) (Figure 3, Table S5). Among the transcriptional regulators, the MarR and PadR families were the most represented; these families often include transcriptional regulators related to the catabolism of aromatic compounds, such as phenolic acids, and, thus, are involved in cell detoxification mechanisms [47]. The high fraction of unique genes represented by a mobilome may reflect the niche adaptation of the different strains that occurred by acquiring or losing specific metabolic abilities based on the nutritive sources and conditions of the surrounding environment. A previous computational analysis on *H. salinarum* NRC-1 (isolated from salted animal hides) also revealed its enrichment in IS-elements, highlighting their involvement in the metabolic and regulatory evolution of this halophilic prokaryote [15]. Indeed, other important categories in terms of gene abundance within the food isolates were related to sugar transferases and transporter coding genes (Figure 3, Table S5). Concerning the first category, the highest number of genes encoded a D-inositol-3-phosphate glycosyltransferase (*mshA*, 16 genes) and a glycosyltransferase family 4 protein (15 genes), whereas the transporters category was mainly represented by the AAA family ATPase and ABC transporter ATP-binding proteins. Other abundant genes belonged to the proteases and peptidases category (Figure 3, Table S5), where metalloproteases and aminopeptidases were particularly evidenced. Microbial aminopeptidase enzymes are of great industrial interest both for food and pharmaceutical applications. For instance, they have been widely used as debittering agents and for protein hydrolysate preparation in the food industry [48]. Moreover, their importance has also been reported in the processing of dry-salted fish [49], indicating that halophilic starter cultures can be exploited to increase the free amino acid content, improving the aroma characteristics [50].

A high number of genes was also evidenced in relation to the reductase and dehydrogenase categories, showing SDR family oxidoreductases (17 genes) and sugar-related dehydrogenases in particular (17 genes) (Figure 3, Table S5).

Concerning the unique genes held by the animal-niche isolates, a total of 604 genes were evidenced among which 499 encoded hypothetical proteins (Table S5). Most of the identified genes were related to the DNA modification/replication/transcription regulations category along with sugar transferases and structural proteins (e.g., flagellin and gas vesicles) (Figure 3, Table S5). Gas vesicles are protein-based buoyancy organelles naturally present in photosynthetic and mesophilic bacteria but also in halophilic archaea. Among the latter, *H. salinarum* gas vesicles are the ones that have been largely investigated for biotechnological applications, mostly related to vaccine development and medical diagnostics [25].

The other abundant genes belonged to the transporter and hydrolase categories, specifically revealing the presence of the sulfur carrier protein TusA (four genes) and the hydroxyacylglutathione hydrolase (glyoxalase II, *gloB*, eight genes) (Figure 3, Table S5). The latter genes are involved in the cell glyco-oxidative stress response since glyoxalase II participates in removing cytotoxic strong electrophiles such as methylglyoxal, an isomer of dihydroxyacetone phosphate that can be formed from the glycolytic pathway [51].

In relation to the environmental-niche isolates, a total of 420 genes were shown among which 344 encoded hypothetical proteins (Table S5). As for the other two ecological niches, most of the identified unique genes were related to the DNA modification/replication/transcription regulations category (sixteen genes) (Figure 3, Table S5). The second most abundant category was then represented by transporters (eight genes), showing amino acid permeases, inorganic phosphate, and HlyC/CorC family transporters in particular (Figure 3, Table S5). This last family of transporters includes prokaryotic Mg^2+^ transporters that, together with Pi uptake, can be used by *Halobacteria* such as *H. salinarum* to form insoluble magnesium phosphate in case of Pi-limited environments [52]. This indicates a key role of some prokaryotes in phosphorus circulation within a specific ecological niche.

#### 3.2.1. Functional and Protechnological Genes in *H. salinarum* Food Strains

Specific metabolic pathways were investigated in more depth with the Comparative Pathway tool in BV-BRC to highlight the putative single-strain capabilities among the food isolates. In particular, the putative ability to produce functional and protechnological compounds was evaluated, and the main outcomes are presented in Table 2.

The different strains showed complete pathways for the production of vitamins and cofactors. Specifically, *H. salinarum* UC4243, isolated from sausage casings, was the only strain including a GTP cyclohydrolase 1 coding gene (EC 3.5.4.16), and it was included in multiple copies (five). This gene converts GTP to 7,8-dihydroneopterin 3′-triphosphate, and it is included in the folate (B9 vitamin) biosynthesis pathway for which the strain was enriched in three other important genes: 6-carboxy-5,6,7,8-tetrahydropterin synthase (EC 4.1.2.50), 7-carboxy-7-deazaguanine synthase (EC 4.3.99.3), and 7-cyano-7-deazaguanine synthase (EC 6.3.4.20). Folate has a key role in different metabolic reactions, such as DNA/RNA biosynthesis and amino acid interconversion. Moreover, this compound has antioxidant properties [53]. Folic acid derivatives (e.g., polyglutamates) are naturally occurring in foods, whereas folic acid is the chemically synthesized form generally used for food fortification and nutritional supplements [54]. Therefore, the selection of food-grade folate-producing microorganisms adapted to high salt levels could be of great interest for increasing the natural food presence of folate in salted food products.

**Figure 4:**
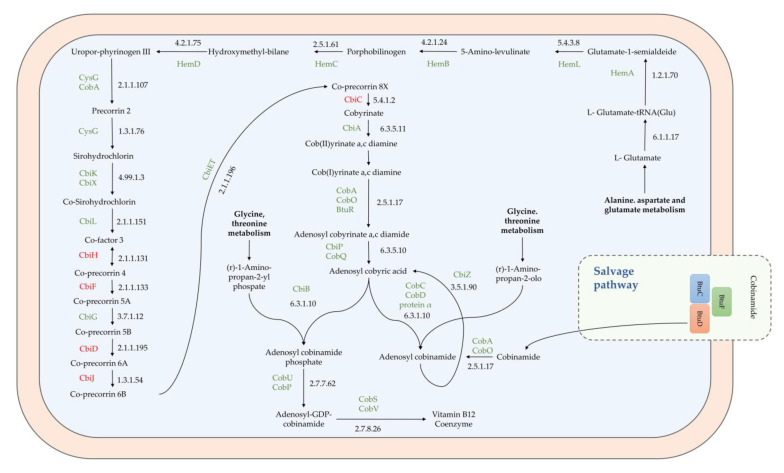
Vitamin B12 coenzyme pathway. Genes present in the food-isolated *H. salinarum* strains are highlighted in green, whereas, in red, absent genes are evidenced based on the general KEGG porphyrin pathway.

*H. salinarum* UC4242, also isolated from sausage casings, presented genes, which were present in multiple copies, coding for ketol-acid reductoisomerase (NADP(+)) (EC 1.1.1.86), acetolactate synthase (EC 2.2.1.6), and dihydroxy-acid dehydratase (EC 4.2.1.9). These genes are included in pantothenate (B5 vitamin) and CoA biosynthesis. Moreover, five out of ten food strains (UC4243, UC4241, UC4236, UC4238, and UC4239) presented a complete pathway for the production of the K2 vitamin menaquinone (menABCDEFG gene cluster) (Table 2). Interestingly, five strains (UC4243, UC4241, UC4242, UC4237, and UC4238) showed an almost complete pathway for vitamin B12 coenzyme production (Figure 4).

The lack of a few genes (indicated in red in Figure 4) involved in precorrin’s conversion to cobyrinate, based on the general porphyrin KEGG pathway, could be due to the partial genotypic knowledge on this biosynthetic pathway in Haloarchaea. However, the presence of a salvage pathway for cobinamide acquisition and de novo B12 coenzyme production has been previously evidenced in *H. salinarum* NRC-1 [55,56,57]. To this extent, *H. salinarum* strains that are naturally present in salted food can be exploited as starters for the production of fortified food products. Indeed, vitamin-fortified food with food-grade bacteria has been largely investigated to improve foods’ nutritional value and, thus, diet vitamin intake in a cost-effective scenario [53,54,58,59,60].

Considering aroma-related compounds, acetolactate synthase (EC 2.2.1.6) was only present in *H. salinarum* UC4242 and in four copies. Within the butanoate metabolism, 2-acetolactate is the precursor of 2-acetoin, an important flavoring agent responsible for the buttery taste of various fermented milk products [61]. The same strain also had a GDP-L-fucose synthetase (EC 1.1.1.271) that converts GDP-4-oxo-6-deoxy-D-mannose to GDP-L-fucose within the fructose and mannose metabolism. L-fucose (6-deoxy-L-galactose) is a rare monosaccharide in nature, but its considerable physiological functions (i.e., anticancer, antiallergic, anticoagulant, and antiaging) have increased interest in the food, cosmetic, and pharmaceutical sectors [62]. Fucose-containing polysaccharides (FCPs) and fucose-containing oligosaccharides (FCOs) can be produced through the fermentation of specific bacterial strains, allowing the exploitation of the different structures of fucose-containing exopolysaccharides (FcEPS). Halophilic bacteria have been previously utilized for the production of FcEPS, and evidence on EPS production and biofilm formation among Haloarchaea has also been shown [25,63,64]. EPS have many applications in the food industry since they can be exploited as thickeners, emulsifiers, and stabilizers to improve the texture, rheological properties, taste, and appearance of food products [63,65,66].

*H. salinarum* UC4242 also exclusively included a gene coding for the inositol-1-monophosphatase (EC 3.1.3.25) responsible for myo-inositol (MI) production. Additionally, this compound is of great interest to the food sector as a nutritional supplement besides cosmetic and pharmaceutical industry applications. Indeed, MI is a polyol that is naturally present in animal and plant cells, and different foods are rich in this compound, such as cereals, legumes, nuts, seeds, and oil [67]. In eukaryotic cells, MI participates in the transduction of several endocrine signals, including follicle stimulating hormone (FSH), thyroid stimulating hormone (TSH), and insulin. Thus, its important role in hyperinsulinemia reduction and ovarian function restoration has been suggested [68,69].

Regarding antimicrobial agents, archaeocins were found in all the strains investigated. Particularly, halocin-C8 and sactipeptides were highlighted. Halocin-C8 belongs to the archaeocins whose activity is not growth-associated, also remaining constant during the cell stationary phase [27]. This halocin is salt-independent, thermostable (up to 100 °C), and trypsin- and organic-solvent-resistant, and its desalted form at −20 °C maintains its activity for more than 1 year [70,71]. Despite halocins generally targeting producers’ closely related haloarchaea species, the inhibition of different species or even of domains has been highlighted [27]. Sactipeptides (sulfur-to-alpha carbon thioether cross-linked peptides) belong to the ribosomally synthesized and posttranslationally modified peptide (RiPP) class of antimicrobial compounds that are found in all three domains of life and that exhibit a huge variety of structures and activities [72]. Besides their antimicrobial effect, these peptides are involved in biofilm formation by improving the adhesion and protection of the producer [73,74,75]. Their involvement in the ability of extremophilic archaea to survive in hypersaline or high-temperature environments has also been suggested [76].

#### 3.2.2. Genes of Concern in *H. salinarum* Food Strains

To consider any possible application of the strains investigated, an evaluation of the presence of potential genes of concern had to be carried out. The main outcomes are presented in Table 3. The high presence of annotated hypothetical proteins within the archaeal genomes indicates, on one hand, that the knowledge of these microorganisms has yet to be explored and, on the other hand, that most of the curated database for the evaluation of such genes are based on the Bacteria and Eukarya domains [77]. However, partial genome annotations of the *H. salinarum* strains can provide useful insights into the possible presence of genes of concern in relation to the salted foods under study.

Genome mining for putative biogenic amine production was performed on the ten food isolates. All the strains (except UC4236) included pyruvoyl-dependent arginine decarboxylase (EC 4.1.1.19) and agmatinase (EC 3.5.3.11) coding genes responsible for putrescine production from arginine (Table S5). The presence of these two genes in haloarchaeal genomes has already been evidenced, suggesting their requirement for archaeal nucleosome maintenance in high-temperature niches [78,79]. In addition, all the strains had an L-tyrosine decarboxylase (EC 4.1.1.25) involved in tyramine production (Table S5). However, up to date, no phenotypic evidence of *H. salinarum* biogenic amine production in food has been highlighted. On the contrary, a previous study detected a reduced or unaltered histamine content during the fermentation of salted anchovies and fish sauce inoculated with *H. salinarum*, suggesting a microbial competition of the species with other histamine-producing microorganisms [50,80].

Regarding ARGs, the interrogation of the CARD database revealed the presence of only one “strict hit” potentially involved in antibiotic resistance in all the strains (except CECT 395). Specifically, the qacG gene coding for a small multidrug resistance (SMR) antibiotic efflux pump was found. This efflux pump is specifically involved in prokaryotes’ excretion of quaternary ammonium compounds used as disinfecting agents and antiseptics. The presence of a small multidrug resistance gene in *H. salinarum* has been previously highlighted (*hsmR*), also indicating its location on the chromosome [81]. 

With regards to VFs, in silico screening conducted with the Abricate tool did not detect significant homologies. The sequence similarity analysis was based on the EFSA cutoff values when evaluating the presence of genes of concern in microorganisms intentionally used in the food chain [82]. However, an additional search of genes putatively related to VFs was carried out with Prokka annotation (Table S5). For instance, genes coding for flagella and pili components were evidenced. Specifically, the flgA1 and flgA2 genes (encoding for the production of flagellin) and the flagellar proteins E and G were found. These proteins play a central role in the formation of the flagellar structure [83]. In addition, several genes were found encoding proteins involved in pili formation, such as prepilin peptidase and type IV pilin [84]. Within the Bacteria domain, flagella and pili are usually recognized as virulence factors since they improve the capability of the microorganism to migrate and anchor to biotic or abiotic surfaces [85]. Moreover, the presence of a gene coding for the IucA/IucC family siderophore biosynthesis protein was highlighted. The production of siderophores is a mechanism that microorganisms, including halophilic archaea, adopt to ensure a constant supply of iron [86]. Nevertheless, pathogenic bacteria and some fungi exploit siderophores to sequester iron and outcompete host uptake during infection [87,88,89]. However, a previous work based on phenotypic tests showed that halophilic archaea belonging to *H. salinarum* do not produce siderophores [90].

## 4. Conclusions

This study elucidated the presence of unique gene clusters of *H. salinarum* strains related to the different ecological niches of isolation (salted foods, animal hides, and solar saltern sediments).

The genome mining of the food strains revealed that *H. salinarum* UC4242, isolated from sausage casings, was the strain with the most food-related protechnological genes (putative biosynthesis of nutritional value compounds, such as B vitamins and *myo*-inositol, aroma- and texture-related features, and antimicrobial compounds). Moreover, all the food isolates investigated presented archaeocins coding genes for halocin-C8 and sactipeptides.

A few genes of concern potentially involved in biogenic amine production (putrescine and tyramine), one gene coding for antibiotic resistance (small multidrug resistance efflux pump), and flagellar/pili protein coding genes were evidenced.

Further phenotypic assessments will be needed to prove both the beneficial and harmful gene capabilities. 

This in silico characterization of *H. salinarum* strains can be used as a starting point for fermentation trials of inoculated salted food to better understand the metabolic potential of these strains and their role within the food endogenous microbial community.

## Figures and Tables

**Table 1:** *H. salinarum* strains investigated in this study.

Strain	Isolation Source	Isolation Category	Genome Source
CECT 395 ^1^	salted codfish	Food	This study
UC4235	salted codfish		
UC4236	salted codfish		
UC4237	salted codfish		
UC4238	bacon		
UC4239	bacon		
UC4240	bacon		
UC4241	sausage casing		
UC4242	sausage casing		
UC4243	sausage casing		
DSM 3754^T 2^	salted cowhide	Animal	NCBI
DSM 669	salted buffalo hide		
DSM 671	salted hide		
NRC-1	salted hide		
AS2	solar saltern sediment	Environmental	NCBI
AS8	solar saltern sediment		
AS11	solar saltern sediment		
AS19	solar saltern sediment		
CBA1132	solar saltern sediment		

^1^ Spanish Type Culture Collection. ^2^ *Halobacterium salinarum* type strain.

**Table 2:** Functional and protechnological genes in *H. salinarum* food strains.

Functional and Protechnological Categories	Gene Annotation	CECT 395	UC 4235	UC 4236	UC 4237	UC 4238	UC 4239	UC 4240	UC 4241	UC 4242	UC 4243
Folate biosynthesis	GTP cyclohydrolase I (EC 3.5.4.16)	0	0	0	0	0	0	0	0	0	5
	6-carboxy-5,6,7,8-tetrahydropterin synthase (EC 4.1.2.50)	1	1	1	1	1	1	1	1	1	6
	7-carboxy-7-deazaguanine synthase (EC 4.3.99.3)	1	1	1	1	1	1	1	1	1	6
	7-cyano-7-deazaguanine synthase (EC 6.3.4.20)	1	1	1	1	1	1	1	1	1	6
Pantothenate and CoA biosynthesis	Ketol-acid reductoisomerase (NADP(+)) (EC 1.1.1.86)	0	0	0	0	0	0	0	0	2	0
	Dihydroxy-acid dehydratase (EC 4.2.1.9)	0	0	1	0	0	0	0	0	2	0
	Acetolactate synthase (EC 2.2.1.6)	0	0	0	0	0	0	0	0	4	0
Menaquinone biosynthesis	Isochorismate synthase (EC 5.4.4.2)	1	2	2	1	1	2	2	2	1	1
	2-succinyl-5-enolpyruvyl-6-hydroxy-3-cyclohexene-1-carboxylic-acid synthase (EC 2.2.1.9)	1	2	2	1	2	2	2	2	1	1
	O-succinylbenzoate synthase (EC 4.2.1.113)	0	1	1	2	1	1	1	1	1	1
	O-succinylbenzoic acid–CoA ligase (EC 6.2.1.26)	0	1	1	1	1	1	1	2	1	1
	Naphthoate synthase (EC 4.1.3.36)	1	1	1	1	1	1	1	1	1	1
	1,4-dihydroxy-2-naphthoate polyprenyltransferase (EC 2.5.1.74)	0	1	1	1	1	1	1	1	1	1
	Demethylmenaquinone methyltransferase (EC 2.1.1.163)	0	0	1	0	1	1	0	1	0	2
Vitamin B12 coenzyme ^1^	Cob(I)alamin adenosyltransferase (EC 2.5.1.17)	2	2	2	2	2	2	2	2	2	2
	Cobalamin synthase (EC 2.7.8.26)	0	0	0	1	1	0	0	2	1	2
Aroma and texture	Acetolactate synthase (EC 2.2.1.6)	0	0	0	0	0	0	0	0	4	0
	GDP-L-fucose synthetase (EC 1.1.1.271)	0	0	0	0	0	0	0	0	1	0
Polyol biosynthesis	Inositol-1-monophosphatase (EC 3.1.3.25)	0	0	0	0	0	0	0	0	1	0
Antimicrobial compounds	Halocin-C8	2	1	1	1	1	1	1	1	1	1
	Sactipeptide	1	1	2	1	1	1	1	1	1	1

^1^ Complete list of genes reported in Figure 4.

**Table 3:** Genes of concern in *H. salinarum* food strains.

Categories of Concern	Gene Annotation	CECT 395	UC 4235	UC 4236	UC 4237	UC 4238	UC 4239	UC 4240	UC 4241	UC 4242	UC 4243
Biogenic amines	Pyruvoyl-dependent arginine decarboxylase (EC 4.1.1.19)	1	1	0	1	1	1	1	1	1	1
	Agmatinase (EC 3.5.3.11)	1	1	1	1	1	1	1	1	1	1
	Tyrosine decarboxylase (EC 4.1.1.25)	1	1	1	1	1	1	1	1	1	1
Antibiotic resistance	Multidrug efflux SMR transporter protein HsmR/QacG	0	1	1	1	2	2	1	1	1	1
Putative virulence factors	Flagellin (flgA1, flgA2)	4	5	7	6	6	6	7	2	5	6
	Flagella E	1	1	1	1	1	1	1	1	1	1
	Flagellar protein G	2	2	2	2	2	2	2	2	2	2
	Putative flagella-related protein H (FlaH)	0	0	0	0	0	0	0	1	1	0
	Type IV pilin	1	3	4	1	2	2	1	1	1	1
	Prepilin peptidase	1	1	1	1	1	1	1	1	1	1
	Flp pilus assembly complex ATPase component (TadA)	1	1	1	1	1	1	1	1	1	1
	IucA/IucC family siderophore biosynthesis protein	2	2	2	2	2	2	2	2	2	2

## Data Availability

Halobacteria 16S rRNA gene sequences from the molecular fingerprinting results have been deposited to GenBank with the accession numbers OQ423240-OQ423272. The whole genome sequencing project for the *H. salinarum* strains has been deposited to DBJ/ENA/GenBank under the project number PRJNA925319 and the accession numbers JAQMIA000000000-JAQMIJ000000000.

## Supplementary Materials

The following supporting information can be downloaded at https://www.mdpi.com/article/10.3390/microorganisms11030587/s1: Figure S1: RAPD-PCR profiles of the 65 Halobacteria isolates from the 3 salted foods evaluated in this study. Figure S2: Roary boxplot representing the number of new genes that were continuously added to the pangenome for each additional genome of *H. salinarum* considered in this analysis. Table S1: The 16S rRNA gene sequence identification of the 33 unique strains resulting from RAPD-PCR fingerprinting analysis. Table S2: General genomic features of the 19 *Halobacterium salinarum* strains investigated. Table S3: Genome-based taxonomic identification of the *H. salinarum* strains analyzed in this study using TYGS tool. Table S4: Distribution of gene categories within *Halobacterium salinarum* pangenome investigated on 19 strains using Roary. Table S5: Gene “presence/absence” of the 19 *Halobacterium salinarum* strains investigated using Roary.
